# Examining the roles and relationships of actors in community health systems in Nigeria through the lens of the expanded health systems framework

**DOI:** 10.1136/bmjgh-2023-014610

**Published:** 2024-10-21

**Authors:** Aloysius Odii, Enyi Etiaba, Obinna Onwujekwe

**Affiliations:** 1 Health Policy Research Group, College of Medicine, University of Nigeria, Enugu-Campus, Enugu, Nigeria; 2 Sociology & Anthropology, University of Nigeria, Nsukka, Enugu, Nigeria; 3 Health Administration and Management, University of Nigeria, Enugu-Campus, Enugu, Nigeria

**Keywords:** Health policies and all other topics, Health systems, Qualitative study

## Abstract

**Background:**

Community health system (CHS) exists through the actions and activities of different actors within and outside communities. However, these actors, their roles and their relationships with one another have not been properly explored to understand their dynamics in facilitating the effectiveness of CHS. This study identified the actors in CHS, described their roles and their relationships with one another using the expanded health systems framework (EHSF).

**Methodology:**

Data were collected using qualitative tools in three states located in three geographical zones in Nigeria. A total of 102 in-depth interviews and focus group discussions sessions were conducted, recorded and transcribed. The respondents were categorised into policy-makers, programme managers, formal health providers, informal health providers (IHPs), civil society organisations/non-governmental organisations, community leaders and community groups. The data were analysed using a thematic data analysis approach.

**Findings:**

The study identified numerous informal health actors (IHA) within the CHS and certain actors—such as community leaders, ward development committees, IHPs and local health representatives—exhibited more pronounced actions. They were active across the EHSF, especially in leadership and governance, health workforce, service delivery and supply of medical products. The relationships and interdependencies of these actors manifest as intricately complex, united by the shared goal of enhancing health at both the household and community levels. Although their roles may not be distinctly defined, instances of active and pronounced engagement reveal the strong commitment of IHA to advocate for and facilitate health programmes at the community level.

**Conclusion:**

There is a broad spectrum of actors whose contributions are critical to the effectiveness and full functioning of CHS. Continuous engagement and defining clear roles and responsibilities for these actors could contribute to improved community health.

**WHAT IS ALREADY KNOWN ON THIS TOPIC**

- The community health system (CHS) is sustained by the efforts and engagements of diverse actors both within and beyond the community.
- These actors contribute to the planning, organisation, coordination and proper oversight of health programmes and interventions to achieve the desired health outcomes.

**WHAT THIS STUDY ADDS**

- The CHS is an intricate network with numerous contributors actively producing, advocating and supporting health at both the household and community levels.
- Within the CHS, the roles of ward development committees, health providers and local health representative stand out prominently and exhibit high levels of activity throughout the expanded health systems framework, particularly in leadership and governance, health workforce, service delivery and the provision of medical products.
- Some actors are only engaged when the need arises; for example, philanthropists, households and community organisations become involved only to address notable challenges.

**HOW THIS STUDY MIGHT AFFECT RESEARCH, PRACTICE OR POLICY**

- Policy-makers and programme implementers must duly acknowledge key players in the health sector and articulate their roles and responsibilities clearly when designing interventions.
- Acknowledging and properly engaging actors in the CHS can significantly contribute to the development of a health system that is more resilient and responsive.

## Introduction

 Communities are the bedrock of any well-functioning health system because they accommodate the consumers of health services.^1^ The significance of robust community health systems (CHS; see online supplemental material 1 for the glossary of terms) cannot be overstated, as they play a vital role in ensuring the health security of individuals and the achievement of universal health coverage. A functioning CHS guarantees that community health providers provide both preventive and curative care and that communities are empowered to demand accountability from governments and service providers in delivering high-quality healthcare.^2^

In alignment with this perspective, the Nigerian health policy emphasises the strengthening of CHS, highlighting the pivotal role of community structures such as ward development committees (WDCs), village health committees (VHC) and health facility management committees in fostering community participation in health.^3^ Additionally, the national strategic health development plan (NSHDP) recognises the need to strengthen and harmonise community-based health providers and other private health sectors such as patent and proprietary medicine vendors (PPMV), drug shops and complementary and alternative health practitioners.^4^ We have categorised these entities as informal health actors (IHA), encompassing a diverse array of functions including service provision, demand creation, monitoring of health services, community mobilisation and participation in programme implementation, among other roles. Despite the existence of IHA, there is a prevailing issue of empowerment. Often, they are unable to fully execute their mandates within the community, resulting in inadequate involvement in the design and planning of health interventions. Consequently, communities struggle to hold the government and service providers accountable.^5 6^

The 1986 Ottawa Charter for Health Promotion recognised the need to engage non-state actors along with governments in health promotion activities.^7^ This sentiment has been echoed in the recent Astana Declaration on Primary Healthcare (PHC), which advocates for the involvement of individuals, families, communities and civil society in shaping policies and plans that impact health.^8^ This collective engagement aims to enhance community ownership and contribute to the accountability of both public and private sectors, ultimately fostering healthier lives for communities.

To meet this demand, there is a need for a more nuanced understanding and application of the concept of CHS to enhance community involvement in health and development, ensuring improved provision and demand for health services at the community level. CHS, as a concept, is continually evolving and has been defined as ‘the set of local actors, relationships and processes engaged in producing, advocating for and supporting health in communities and households outside of, but existing in relationship to, formal health structures.’^9^

CHS, as a concept, is highly contextualised. It has been framed as the space between the ‘local health system’ and the community, filled by the community health workers who form a critical component of the CHS acting as service extender, cultural broker and social change agent, extending health services to households.^10^,^12^ Additionally, it has been conceptualised as the ‘grey zone’ between the public health system, non-governmental organisations (NGOs) and private health system.^13^

However, the challenge lies in the often invisible nature of many actors within CHS, including community groups, informal health providers (IHPs), faith organisations, sporting groups, social networks and non-governmental sectors.^14^ Despite their significant contributions to healthcare in the community, these actors are frequently overlooked and not well-characterised in the literature.^13^

From a systems lens, there are many actors and players, working with strong community support, that drive the CHS. The existence of many actors interfacing between the formal and informal structures at the community level whose roles influence healthcare delivery has been acknowledged.^11 15 16^ These actors ensure that health programmes and interventions are well-planned, organised, coordinated and properly controlled to meet desired results.^17^ However, the IHA roles and relationships are often disregarded, presenting a substantial gap in our understanding of the holistic CHS.

The implications of this oversight are substantial, as it neglects the interconnectedness of these IHA with each other and with formal health actors (FHAs).^9^ Acknowledging and mapping the full array of health actors within the CHS, their roles and relationships is crucial. This not only recognises their contributions to community-level health but also informs future engagements and underscores their influence on change, connected to political and economic contexts.^18^

Therefore, this study aims to map the key actors in the CHS and describe their roles and relationships with formal and IHA. This will offer new knowledge to health system researchers, policy-makers and programme implementers of the intricate dynamics within CHS. It will also contribute to the growing literature on ways to strengthen CHS and how to harness resources at the community level to improve health security of local community members, especially in combating infectious diseases and for health promotion.

## Materials and methods

### Conceptual framework

The conceptual framework of the study is based on the expanded health systems framework (EHSF). The WHO developed six (service delivery, health workforce, health information system, medicine and technologies, finance and leadership/governance) components that are essential for the effective functioning of health systems.^19^ Sacks and their associates argued that there is a need to expand the framework to capture much of the health practices by community health actors taking place in homes and community level.^20^ The expanded framework now captures household production of health, social determinants of health, community organisations, service delivery, health workforce, medical products, vaccines and technology, social partnerships, financing, leadership and governance, and information, learning and accountability.

Actors in the CHS were organised into categories developed by Schneider and Lehmann.^9^ The authors captured these actors as including (1) household caregivers; (2) providers (formal, informal and volunteers working in the community); (3) organisational intermediaries (4) government sectors such as housing, education and social development and (5) representatives of local health and political structures. This broad categorisation was broken even further to isolate those making any contributions, including those playing minor roles.

We fit the descriptions of actors, their roles and relationships within the EHSF. This became imperative due to the significance of these insights for national and global policy-makers, aiding them in allocating resources strategically to facets of the health systems that foster community health.^20^

#### Study design and area

The study adopted the qualitative descriptive research design. This design is suitable when the study does not require a deeply theoretical context, and the research intends to stay close to participants’ experiences or provide a straightforward description of participants’ perceptions and experiences.^21 22^ This approach is recommended when researchers ‘want to know, regarding events, who were involved, what was involved and where did things take place.’^23^

The study was undertaken in three states, selected across three out of the six geopolitical zones in Nigeria. One state each was purposively selected from North-west zone, South-south zone and South-east zone. Each state had some evidence of present or past community health initiatives. The three states were selected from different zones to achieve a geographical representation of the country. In each state, two local government areas were purposively selected, each representing rural and urban communities.

#### Study participants

The study population was drawn from various health-related sectors, each contributing directly or indirectly to community health programmes. This population was categorised into the following (see table 1): policy-makers, programme managers, formal health providers (FHPs), IHPs, civil society organisations (CSO)/NGO, community leaders, and community groups. The community groups include community members (men/women) and other voluntary groups. The aim was to gain as many diverse views as possible and to capture those who may have been excluded in related studies.

**Table 1:** Distribution of participants

Categories	States		
	South-south zone	South-east zone	North-west zone
Health sector Policy-makers	Director of health (2) Ass. director of works	MoH (2)	HoD health (2) LG engagement officer
Health programme managers	Coordinator, community social development project State nutrition officer	Community coordinator/mobiliser Leader, National Youth Council Drug Programme	Supportive supervision programme manager
Formal healthcare providers	Lab. technician OIC (2) Pharmacy technician	OIC (3) Private medical doctor Midwife, private maternity home (2) Private pharmaceutical sales rep	OIC (2) Matron Deputy OIC
Informal healthcare providers	Traditional birth attendants (TBAs)- (4) Traditional bonesetters (2) Herbalists (2) PPMV (5)	Native doctor (2) Lab. scientist, Chinese trained, unregistered TBA PPMV (2) Traditional bonesetter	PPMV (3) Bone setter (2) Tooth expert Traditional healer Herbalists TBA (3)
CSO/NGO	FHI 360 Red Cross	Director, local CSO Coordinator, local CSO CSO coordinator (2)	Chairman, farmers’ association Malaria consortium Georgetown global health
Community/religious leader	WDC chairman Health facility committee chairman Woman leader (2) Village head (2) Refugees leader-1	WDC chairman (2) Community leader (2) Religious leader	WDC chairman (2) WDC treasurer Ward head Village head Religious leader (2)
Community groups	FGD with women’s group (2) FGD with men’s group (2)	FGD with women’s group (2) FGD with men’s group (2)	FGD with women’s group (2) Voluntary community mobilisers (3) FGD with men’s group (2)
Male/females	18/17	18/13	25/11
Total Interviews per State	35	31	36

CSO, civil society organisation; FGD, focus group discussion; FHI, family health initiative; NGO, non-governmental organisation; OIC, officer in charge; PPMVs, patent and proprietary medicine vendors; WDC, ward development committee.

The mapping of actors was guided by literature and informal discussions with community mobilisation officers (CMO) who served as contact persons. A total of 102 in-depth interviews (IDIs) and focus group discussion (FGD) sessions were held with diverse stakeholders, with the breakdown as follows: policy-makers (8) health programme managers (5) FHP (15) IHP (31) CSOs/NGOs (9), community leaders (19) and community groups (15). Overall, 90 IDI (policy-makers, programme managers, FHP, IHPs, CSO/NGO, community leaders) and 12 FGD sessions (community groups—women and men groups) were held with the participants.

#### Instruments and data collection

The pretested data collection instrument was developed by researchers with years of experience in health systems research. The CMOs helped to mobilise the participants by informing them of the nature of the research and the risks and benefits. They also assisted in distributing a letter of introduction, containing the informed consent, to respective officers. All respondents provided informed consent before they were interviewed. Community leaders were interviewed within their communities, while policy-makers and programme offices were interviewed at their offices and other workplace environment. Community groups, FHP and IHP were interviewed at the PHC centres while the CSOs/NGOs were interviewed in their offices.

The language of interviews was English and native languages (Hausa, Igbo and Ibibio) depending on the state where the interviews were conducted. The interviews in Igbo, Hausa and Ibibio were conducted by data collectors who are fluent in these languages. Their training included reflection and consensus-building on the interpretation of concepts and phrases in the tools, conducted in the participants’ native language. Interviews lasted an average of 47 min for the IDI and 1 hour for the FGDs.

#### Data analyses

The data from the interviews conducted in English were transcribed verbatim. Interviews in native languages were transcribed in the native languages and then translated into English Language by experts in the native languages. In qualitative descriptive research, codes emerge directly from the data, reflecting its purely data-derived nature. We employed the coding reliability approach to integrate each code into our framework effectively. The coding reliability approach is a type of thematic analysis that involves early theme development and coding involves identifying evidence for themes.^24^ First, broad themes from the extended health systems building framework were noted. Then a structured approach was used, using a codebook developed collaboratively by the team. Two members of the team worked independently to develop a codebook using predefined transcripts. The codebook was refined through repeated discussions by members of the team. The use of multiple codes led to a level of agreement between coders, leading to consensus on the final coding framework. The codebook was organised using Excel files.

#### Reflexivity

In our use of the EHSF, we were mindful of the theoretical underpinning and the need for a comprehensive view of community health practices. However, our interpretation and application of the EHSF may have been influenced by our own perspectives and disciplinary backgrounds, which could impact the analysis and findings. Choosing a qualitative descriptive approach for study design and area selection was deliberate, based on our research goals and practical considerations. This choice reflects our judgement about the most appropriate methodology for addressing the research questions, but this may have also been shaped by our prior experiences and preferences. Collaboration with CMOs facilitated participant engagement. We recognise the potential power dynamics between researchers, CMOs and participants, which could influence the research process. We reflected on our interactions with participants and considered how these dynamics may have affected their participation in the research and the data collected. In data analysis, we used a structured coding approach but were aware of our subjectivity. The use of multiple coders helped us manage this by measuring the level of agreement among coders. Reflexivity helped mitigate bias and enhance the credibility of our findings.

## Results

### Mapping of stakeholders in the CHS

Stakeholders were classified broadly based on the following: household-level caregivers, providers (formal, informal and volunteers), organisational intermediaries, government sectors, private individuals and local health representatives (LHRs). Under each category, actors were grouped based on the uniqueness of their roles and how they distinguished themselves in terms of their contributions to the CHS (see online supplemental material 2). For example, WDCs who were classified under organisational intermediaries were given special attention because they were major contributors.

### Roles and relationships among actors in the expanded health system framework

#### Health workforce

The health workforce in the CHS can be classified into formal and IHP. While the likes of midwives, CHEWs, community health officers (CHOs), nurses, medical doctors and laboratory technicians are the formal providers, the IHPs include traditional birth attendants (TBAs), bonesetters, herbalists, community health influencers and promoters and PPMVs. Formal healthcare providers are engaged in PHCs and must have been certified by the PHC board to provide health services. The challenge, however, is that they are hardly sufficient to serve the host community.

Community leaders, exemplified by the WDCs and village heads, play a crucial role in addressing the critical issue of human resource gaps in PHCs. Their proactive approach, driven by a keen awareness of the healthcare needs within their communities, involves advocating for increased health workers through formal channels. When community leaders observe or are informed by FHPs about a shortage of human resources in PHCs, they take it on themselves to initiate change. This involves composing letters addressed to the local government health authority, a body comprising key figures such as the local government chairman and the head of the department for health. Their correspondence emphasises the urgent need for additional personnel in health facilities to enhance service delivery. Often, responses from the local government health authority fall short of meeting the communities’ requests. For example:

We have only two nurses here. The other ones are these Red Cross people, so I have written up to 5 times to the chairman of the local government to assist us and give us somebody to assist in case something happens when the Officer in Charge (OIC) is not around. But nothing has been done till this time (IDI with Community leader, South-south zone).

Data from the state in North-west zone illustrate the community leaders’ commitment to finding practical solutions to the human resource gap. In the face of unmet demands, the WDC take extraordinary measures by recommending health graduates as potential volunteers to serve in health facilities. For example:

We have volunteers, our people. Most of the students, when they graduate, serve as volunteers before the local government starts paying them. They (WDCs) are the ones who bring their people and say, ‘this one and this one.’ We agree and we stand and they help out. However, they (the volunteers) have to apply through the primary health care coordinator’s office. The facility will not accept them unless they meet our staff… they have to be cleared by the LGA (IDI with HOD, North-west zone).

There were reports of FHP and IHP being trained by the government and in collaboration with WHO, UNICEF and NGOs such as family health initiatives (FHIs). In the south-south zone, medical practitioners often train IHF to update their knowledge on maternal and child health and the use of health technologies. At the end of the training, they are given stipends:

I go to community informant training as well as TBAs training at the health centre that is being organized by the government where expert doctors are brought in to lecture on pregnancy and childbirth and how to go about ensuring the safety of mother and child, including the knowledge about tools to use during this process. I also attend Family Health Initiative (FHI 360) training to broaden my knowledge. Yes, we were given 5000 naira at the FHI training. Both the FHI training and training at health centres teach us how to do these tests (IDI with TBA, South-south zone).

TBAs have also been trained and recruited as volunteers into the formal health system. Additionally, volunteers are recruited by WHO and UNICEF to assist healthcare providers, but more specifically, to help mobilise community members to seek healthcare in health facilities and also participate in health promotion activities such as immunisation. They are compensated with stipends and may receive other incentives from the officer-in-charge of facilities as a way of motivating them. The volunteers may not have a background in health, but they are trained to create demand for the use of health facilities and to serve as intermediaries between the community and the health facilities. The community leaders are heavily involved in their selection as noted by this quote:

They also select people in the community in other to mobilize people (health seekers). As I said, they go through the district head. Mostly, they are female. They mobilize people on how to go for ANC when they deliver, and encourage them to go for immunization (IDI with HOD, North-west zone).

Thus, IHP is in regular contact with FHP as a result of these partnerships. However, this is most notable among those that have been properly engaged by bilateral agencies and state governments as mobilisers. It is crucial to acknowledge, however, that not all IHPs are equally integrated into this collaborative framework. Some practitioners operate independently of government structures, FHPs and bilateral agencies. Despite this autonomy, their contributions to healthcare delivery are notable and deserve recognition.

#### Service delivery

The data revealed that services are delivered in both facility and community settings, driven by FHPs and IHPS in these spaces. Key actors in facility settings include midwives, CHEWs, nurses, medical doctors and laboratory technicians. Besides providing facility-based services, they occasionally go for outreach to the communities for immunisation and health promotion activities. By doing this, they deal directly with household heads and members. However, this must be done with proper engagement with community leaders and the members of the community. As noted in the data, misunderstandings could arise if community leaders and members are not properly engaged in health activities taking place. This is illustrated in the following quote about an environmental health officer:

Yes, then, they (community members) will quarrel with any officer of the village council about why they allowed the sanitary officer to enter their compound, and that no environmental officer has to enter into their house (IDI with Community leader, South-south zone).

Informal healthcare providers include health practitioners who have not undergone formal training. The study identified the following actors as IHP, TBAs, PPMVs and traditional healers (bonesetters, healers and herbalists). Informal healthcare providers are dominant in the community setting, where they provide services using various means (drugs, herbs, other local substances like powder and liquid, and even divination) for the treatment of illnesses. Households patronise these groups when they perceive that their illness is spiritual or that orthodox medicine cannot improve their condition. For illustration:

In some cases, the sick persons’ family members bring the person (to us) when the medical treatment has failed. Then will say, ‘let me know what the problem is maybe it is an ancestral thing that is disturbing the person (IDI with traditional healer, South-east zone).

When a patient is brought to the hospital, they first examine the patient and determine if the sickness can be treated with formal medicine or not, if it cannot be treated, they then transfer the patient to us the spiritual healers, if it is beyond our capacity, we refer them to traditional healers. (IDI with Spiritual healer, North-west zone).

In addition to providing services to households, FHP and IHP also refer patients to one another. However, the findings show that it is more common for referrals to be from IHP to FHP but for different reasons. While IHPs refer patients to FHP when they perceive that the illness is complicated, FHPs refer when they perceive that the illness is best treated locally. The following quotes are illustrative:

There are instances where we refer patients with complicated cases to the hospital for example those that need to have an ultrasound scan (IDI with Bone Setter, North-west zone).

They do it occasionally (refer patients to us), but not always. Some illnesses look like electric ants wrapped from the front of your body to the back and can kill if not treated properly, so I have leaves and herbs that I use to treat such illnesses (IDI with Herbalist, South-south zone).

The data also suggested that IHPs do refer patients to one another. For example, patients who suffer strokes without hypertension might be advised to seek alternative treatments like visiting a church or herbalist. The following quote is illustrative:

If I have a patient who does not suffer BP (*hypertension*) and had stroke, I will not waste the person’s time on drugs, I will tell the person to go to a church or go to an herbalist. Normally any person who has stroke must have a BP, but now if the person has BP and the person has stroke either partial or total, the person will be asked to control the BP, and when it goes down the person will be treated (IDI with Lab operator, South-east zone).

#### Household production of health

Households actively engage in health production by using a range of remedies derived from plants, animals and minerals. Additionally, informal health practices, originating within households and handed down through generations, contribute to household production of health. Also, in many households, self-medication is prevalent, manifesting in the use of locally sourced remedies and the acquisition of medicines from PPMVs to address a variety of ailments. This ingrained practice has become a cultural norm within communities, where individuals often resort to self-medication as a first line of defence against illnesses.

Our people, ab initio, are not threatened with health matters for health matters are very much indigenous with us. To start with, historically the founding father of this community is a great *dibia* (Native doctor). By all indications, it is clear that oriental medicines are with us right from origin (not alien to us). Therefore, our people know something about Mgborogwu na Mkpaakwukwo (traditional herbs and leaves) (IDI with Ministry of Health, South-east zone).

There are scenarios when neighbours step into the role of healthcare providers when the situation demands. However, it is important to note that the reliance on self-medication and informal health practices does not always ensure a positive response to treatment as indicated in this quote:

Like there was a time I was ill, and a woman came and told me to use herbs. She used her hand to cut the leaf for herbs and squeezed it. It looked like blood, she asked me to add one tin of milk to it which I did, she asked me to drink it and I did, not quite long I became sick again (IDI with community leader, South-east zone).

Moreover, households occasionally take the initiative to organise environmental sanitation efforts and construct drainage systems on their own. However, it is more common for such initiatives to be spearheaded by community leaders, who proactively mandate households to undertake the task of clearing their surroundings. This collective responsibility ensures a coordinated approach towards maintaining a clean and hygienic environment. In the state in North-west zone, the community leaders frequently incentivise some community members to construct drainage and the youths participate by supervising the project. Similarly, in the state in Sout-east zone, the state government, through the health and environmental departments contributes by providing waste disposals:

The community, they are the ones that initiated it (drainage). Yes, the community members are the ones doing it (spraying mosquito insecticide and environmental sanitation). They select persons to do it then they pay for it. They are volunteers. There are some people responsible for the general sanitation. They go together with the youths to see what activities that are going on (IDI with CHEW, North-west zone).

Talking about sanitation, during the previous governor’s regime, he provided container to dispose wastes in street (IDI with TBA, South-east zone).

Formal providers such as CHEWs are sometimes contacted and empowered by NGOs to give health advice to households whenever there is a disease outbreak. This was quite pronounced during the emergence of COVID-19.

#### Community organisations

Existing community actors aiding the penetration of community health programmes include community groups like faith-based organisations (FBOs), WDCs, VHC, community leadership (ie, Emirs, district heads, president generals, village heads, traditional leaders and the council of chiefs), women groups, youths and volunteers. Community volunteers like cogroups and voluntary community mobilisers (VCM) go from house to house to mobilise community members to seek healthcare in the health centres. The WDCs and VHC work closely with other community leaders to identify health challenges, call for support and ensure that their members benefit from community health programmes. They could also serve as representatives in health programmes and step down to community members, for illustration:

Yes, I was one of those who participated in that workshop so when I came back, I carried the same information to the village. I summoned the whole general village people and lectured them, based on the same lecture I was given, with my COVID-19 data, up till today I still lecture people (IDI with community leader, South-south zone).

The approaches to community engagement could be top-down, bottom-top or horizontal. For top-down, multinational agencies and governments who intend to pioneer a project may seek community leaders to help create awareness in the community. Community groups such as volunteers usually play critical roles by mobilising those in need of health services. Furthermore, through religious leaders and other FBOs, health programmes are announced in churches and mosques targeting the people who turn up for religious gatherings. For example:

Yes, even yesterday in the church, I announced to the church member of immunization against measles and immunization for Covid-19. In the local community where am serving they come to the church to create awareness, test people and sometimes give people free drugs. There is also an ongoing one for breast cancer check for females at a nearby hospital because early detection can go a long way in solving the problem. (IDI with community leader, South-east zone).

As for the bottom-top, community leaders reach out to the government and other stakeholders to meet their health needs. When it is horizontal, community groups, leaders and her members mobilise themselves to solve their health needs themselves. This could be through asking those well-to-do (philanthropists) or household members to make certain donations as illustrated in this quote:

The community came together to form that activity (sharing pads with school girls), they are the ones that are contributing that money through different people. So, after contributing the money, they select the school and location, usually a very poor environment or locality where you can see girls who cannot buy pads. Rather than allow them to address their period using local methods, they are receiving the pad (IDI with CHEW, North-west zone).

IHPs also helped with organising their clientele base, preparing them to be receptive to formal health interventions. The WDCs and VHCs together with the community leadership are usually at the forefront of receiving providers, monitoring facilities and seeing to success of health programmes in communities.

#### Societal partnerships

Government, organisations and providers partner with community groups to improve the reception and delivery of health services in the communities. It is widely believed that if the community leadership structure does not buy into health programmes, there is a likelihood of failure. So, organisations have been deliberate in setting up community structures such as the cogroups and the VCM to bring about the necessary community presence needed for the penetration of health programmes and interventions. Others rely on existing community structures like health facilities to provide care:

They (Red Cross) came to help refugees from Cameroon, and they decided to use our facility to provide healthcare for the refugees from Cameroon. We have only 3 facilities in the state for the refugees… The refugees are all treated for free in these facilities (IDI with community leader, South-south zone).

Providers in health facilities could play supervisory and coordination roles over community representatives of the health programmes. As part of the partnerships, community-based organisations such as youth organisations and women groups play assistive roles in health facilities, especially in the areas of footing the bills of indigent patients and in sanitation. Other times, they finance the project themselves while partnering with other community groups as illustrated below:

They check BP, sugar, these labs that you can do and take the result as well, but if it involves the eyes, they partner with the Niger Optical, their doctors or medical personnel will come and carry out this exercise. Yes, like Umuada Igbo they were there with us at the flag off day, they identify with us, they even went extra mile to discuss it with the Umuada groups in other clans (IDI with CSO, South-east zone).

#### Medical products, vaccines and technology

Medical products are obtained through the help of state government, multinational agencies and philanthropists in the community. They may include delivery kits, drugs and insecticide-treated nets (ITN), prescription eyeglasses, sugar monitoring equipment and high blood pressure equipment. PHCs also contribute using service charges to procure medical products and other infrastructures that are needed at the centre. These items are usually stored at the health facilities and distributed to community members, using designated persons in the communities. In the North-west zone state, they are overseen by the WDCs:

Sometimes, when there’s an NGO that gives drugs, the government notify us so that we can also have our people in the pharmacy especially the malaria drugs (IDI with WDC chairman, North-west zone).

Also, medical products are distributed by agencies for health but are coordinated by government health agencies like the state Ministry of Health. Additionally, community mobilisers are used to distribute medical products like ITN and over-the-counter medications. Community mobilisers are well known in their communities and usually act as the link between health initiatives and the community. The following quotes are illustrative:

Few weeks ago, through the health centers, they were giving out some drugs, I think river blindness or something like that, they distributed it among the elderly ones and others also. But in x town we have two wards, one forward one, one forward two. So, they were specific people that were given it to so that people will be going to their houses [to collect them] (IDI with LG Community Coordinator, South-east zone).

I don’t know but sometimes, we are asked to inform the mothers to come and collect some certain medication for the children like the albendazole, vitamin A, and they distribute the medication from time to time (IDI with VCM Mobiliser, North-west zone).

#### Health financing

Donors such as WHO, United Nations, UNICEF and humanitarian organisations such as the Red Cross usually step in to fund some health projects in the community. Redcross was said to have funded the treatment of refugees in the South-south zone, through a selected primary health centre. They also sponsor certain treatments referred to secondary health facilities. To ensure transparency and accountability in the execution of these health projects, the Red Cross has taken proactive measures by setting up committees to oversee and supervise the activities of healthcare providers operating within the supported health centre. This strategic approach was to ensure that the intended beneficiaries receive high-quality healthcare services:

We have a committee set up by the Red Cross. So, some of the refugees are represented. So, whatever is happening here, we are in the know when they are going to buy drugs, we are in the know. How much is used to buy drugs, we are in the know (IDI with Community leader, South-south zone).

Data also provided evidence that some health programmes are funded by community members either collectively or individually. These programmes could be preventive, diagnostic or curative. For example:

They have not done anything at all, it is the people at home that are supposed to motivate them to do something, but those at home said the government should renovate the facility and other things, because, the revenue we are generating here, we are handing it over to the government and not to the community (IDI with CHEW, South-east zone).

In the North-west zone state, it was also found that infrastructures in the health centres like benches and the renovation of facilities are bought from the money obtained from the state’s health scheme. Also, it was found that community members contribute to financing health projects through levies. Community leadership structures also support projects using their own money. It was also found that communities do come together to support the health of young girls through the distribution of pads.

Yes! The community came together to form that activity, they are the ones that are contributing their money through different people. So, after they contribute the money, they will select the school and community and location like a very poor environment or locality where you can see girls that cannot buy pads to prevent themselves or to create hygiene in their community. Rather than using pads during their period, they dress themselves using local methods. They receive pads (FGD with men, North-west zone).

Finally, it was found that some private individuals fund healthcare in their communities as a way of promoting community health. It could be in the form of bringing health workers to conduct tests or procuring drugs for community members. The timing and extent of coverage of the programmes are mostly the decision of the private individual.

#### Leadership and governance

Community leadership roles are enormous; summarily, it is about interfacing with state officials and agencies on community health. The programme officers are introduced to the village heads by the community leaders (ie, WDCs). Then the village heads assist in finding locations or halls for the programme and coordinate with town criers, who inform the members of the community. For illustration:

First, the community leaders receive them, not the women, and then take them to meet the village head of the community, who will determine where they will stay to hold these programs and also inform the town crier about this program so men, women, and children can attend (FGD with women, South-south zone).

Community advocacy was carried out by community leaders to politicians, at the local and state governments. In other instances, it could be within the community by someone who has the ear of a local politician. This is done when there is an urgent matter that needs to be solved or addressed.

If I discover that there is something we need that maybe it is difficult to procure, I contribute by going to a politician with a proposal or letter or something to the politician and tell him that the community need him to assist the health immediately and that it should be done immediately. That’s what I contribute (FGD with Men, North-west zone).

There are instances where community leaders are joined by the FHP to embark on advocacy to the government. For example, there is an instance where healthcare providers team with community leaders to influence the decisions of local government authorities for their benefit:

As a team, we went there together with the team of the community group. We went to the chairman (local government administrative head) and explained our challenges that we have a problem with delivery at night, so he gave us that generator because then our big generator that is taking the entire hospital is faulty (IDI with Nurse, North-west zone).

The health facility committee was reported to supervise an aspect of the community health programmes. They work closely with the OICs and state governments to monitor and support projects.

Community leadership structures’ contributions to health are based on passion and the desire to see an improvement in community health. It was learnt that most of them are not paid but participate voluntarily. However, the security situations in some communities are demotivating the leaders from being committed to health issues. For example:

Who will gather people, is it the PG? he still wants to live, and so is his committee, they still want to live or the Oji and his cabinet, they still want to live, especially those living outside the community. Anyone who hears what is happening will run away, nobody wants to buy death, so that is where we are now (IDI with community leader, South-east zone).

#### Information, learning and accountability

There are multiple players within the CHS working to collect information. The actors included CHEWs and community mobilisers. The information collected at the community level includes immunisation and antenatal care visits. The data are mostly collected from pregnant women and children between the age of 0–5 years. The actors included CHEWs and community mobilisers. CHEWs collect data from health seekers at the facility while community mobilisers collect data at the community level.

We collect data during immunization, vaccination. We also keep records of all the patients’ details, those treated in the facility. Even those who were given condoms and mosquito nets (IDI with CHEW, South-east zone).

The community has a limited role in data collection and use, except in information dissemination. Community-based groups like town criers/community PROs and community health advocates help in disseminating health information to the public. When state governments are planning health programmes, they are involved and help to disseminate major decisions to the public:

Yes, I am community health advocate. I’m spreading information anytime government or community organization or NGOs are planning for programs, health program in the community we are the first people to know because of the information. We are the ones that pass information to the district head, say advocacy is planning for this, in certain time and place it will happen (FGD with Men, North-west zone).

## Discussions

**Figure 1:**
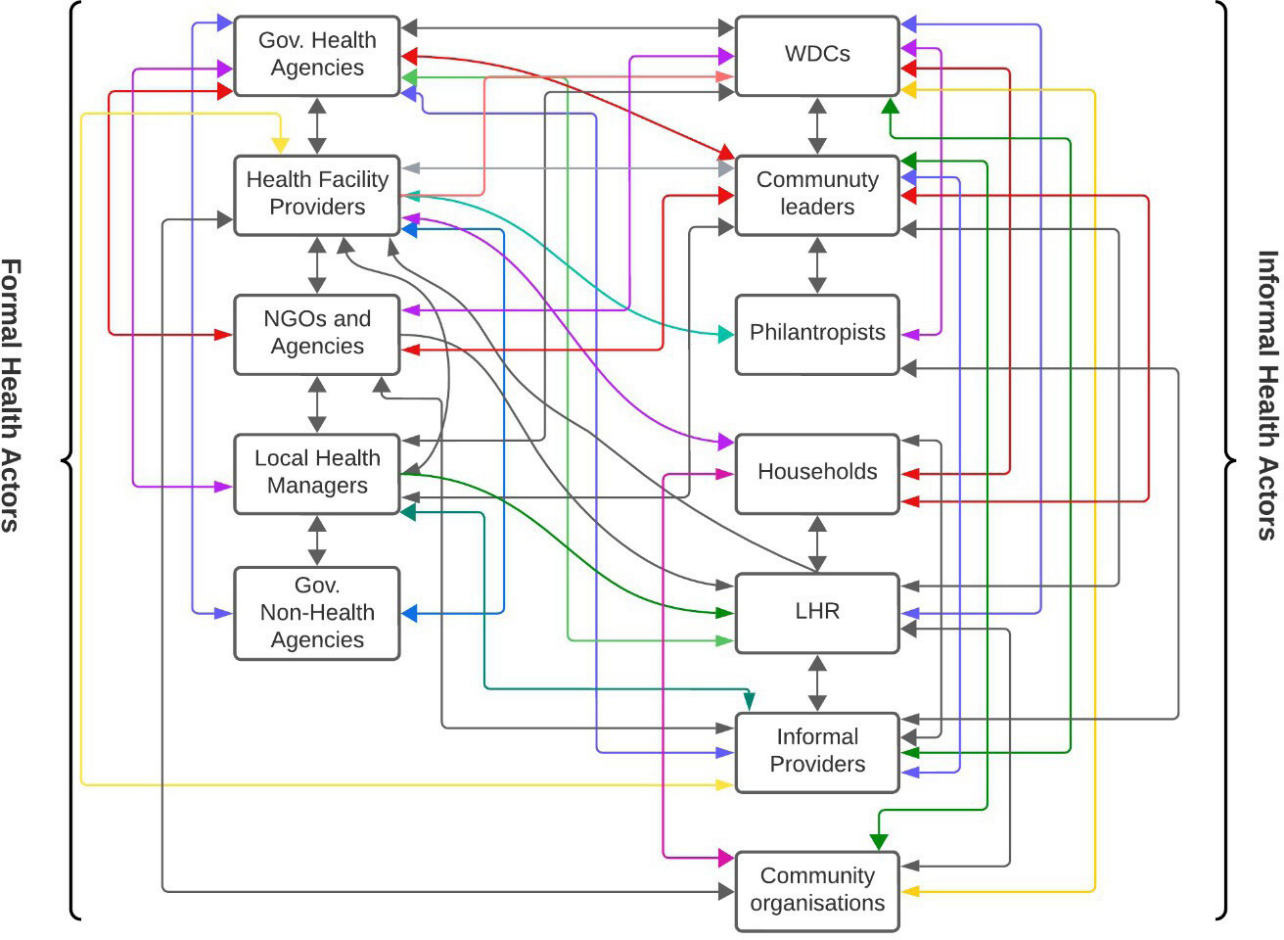
Actors and their relationships with each other. LHR, local health representative; NGOs, non-governmental organisations; WDCs, Ward Development Committees.

The study has shown that the CHS is a complex web with multiple players producing, advocating and supporting health at the household and community level. It was earlier posited that many actors exist in the CHS.^9^ Outside the well-known actors, many hidden players exist in the CHS whose roles and contributions are barely acknowledged. In this study, IHAs were identified, and their roles and contributions to the CHS were described, illustrating their interconnections and contributions to community and household health within the context of the EHSF (see figure 1).

The findings underscore that the presence of diverse actors within the health system significantly improves access to health services. In another study, it was reported that involving multiple actors contribute to increased health information and increased use of health services.^25^ However, this is not the case in some communities where there are reports of the lack of involvement of community actors in community projects.^26^

The findings showed that IHAs contribute in leadership and governance, financing, medical products, information, learning and accountability, service delivery, and the provision of the health workforce. Their roles cut across the entire system without a centralised management structure and a formalised process for engaging with other actors. Nevertheless, they are obligated to conduct themselves in a manner that aligns with the interests and values of the community. Previous study has shown that trust among key actors is paramount for the growth and effectiveness of CHS.^27^

Certain actors only relate to each other when the need arises; for instance, philanthropists, households and community organisations become involved only under specific circumstances. In another study, different community actors rely on networking with system providers to coproduce health services.^28^ But, in this study, sometimes, actors such as WDCs, LHR and IHPs are self-organising, which forms the strength of the CHS. While exploring the potential for a more centralised management structure for these actors, it is crucial to explore its likely consequences.

The study also found that many external organisations and stakeholders influence the make-up and functionality of CHS. For instance, organisational intermediaries’ (WHO, UNICEF and Red Cross) often initiate relationships with providers, government sectors and LHR at the community level for healthcare services. Similarly, the government sector engages with one another and collaborates with IHP and LHR. However, they predominantly dictate health programmes, and the community only operates within the confines of the roles assigned to them. This approach has faced criticism for its susceptibility to challenges stemming from the power imbalance between FHA and community stakeholders.^28^ It was argued for a strategic engagement of actors outside the health sector and this is because many global health challenges are intersectoral.^20^

Furthermore, the study revealed the significant contributions of community leaders, WDCs and LHR in CHS. They act as supervisors and participate in coordinating and mobilising their members for positive health action. In a study in Malawi, community leaders have many roles, including advisory, encouragement, regulating and restricting cultural practices, formulating bylaws and handling sexual abuse complaints.^29^ In this study, due to the importance of community leaders, donor agencies and government officials often engage them when commissioning or delivering health programmes. They are also able to command respect because the community selected them, so they are accountable to them.

### Implications

The findings of this study show that public health interventions, including ones that aim to improve the overall community health status must tap into available community resources. This is considered crucial in resource-constrained settings,^25^ including communities experiencing disasters.^30 31^ As demonstrated in the present study, they are instrumental to the success or failures of any health interventions programmes. However, for this to be effectively done, there must be proper recognition of key players in health and clear communication of their roles and responsibilities. In a study in Australia, community leaders who were not properly engaged were said to block health messages that go against their wishes.^32^

Essential structures that play a crucial role in facilitating relationships in the CHS are the WDCs (when active), community leaders and LHRs. These structures use a systematic approach to interact and cooperate with themselves and with other informal and FHA, fostering effective communication, shared goals and mutual understanding. The pivotal role played by these individuals stands as a testament to the remarkable resilience of community structures in addressing human resource gaps within healthcare and facilitating the provision of essential materials for healthcare delivery. Beyond underscoring their unwavering dedication to the well-being of their communities, their actions emphasise the imperative for collaborative actions between these community actors and formal health structures. This collaboration is crucial for effectively addressing systemic challenges within the healthcare sector. Recognising and supporting these actors can make a substantial contribution to the establishment of a more resilient and responsive healthcare system.

### Study limitation

The study has certain limitations, notably the possibility that not all stakeholders operating within the CHS in all states were identified comprehensively. Additionally, due to the numerous actors included in the study, some of their roles may not have been exhaustively described. Also, given that this is a one-country study, it may not be generalised to other low-income and middle-income countries. This is because actors, their roles and relationships with one another may differ in these settings. Nevertheless, the study provides valuable insights into understanding roles and activities, offering useful information for the benefit of future researchers, policy-makers and implementers.

## Conclusion

The study has revealed the numerous actors in the CHS and explored how interconnected they are to one another. The interconnectedness of various actors has an impact on the overall effectiveness of CHS. Therefore, there is a need to acknowledge these actors and their interconnectedness to one another. Doing this will serve as a pathway for informed decision-making, in terms of ensuring that community actors are properly engaged during health programmes. Policy-makers and formal health authorities can leverage this understanding to formulate policies that empower local leaders and their representatives as this will foster collaborative efforts that drive positive health outcomes.

## Supplementary material

10.1136/bmjgh-2023-014610 online supplemental file 1

10.1136/bmjgh-2023-014610 online supplemental file 2

## Data Availability

Data are available on reasonable request.
