# Supplementary material for: Examining the roles and relationships of actors in community health systems in Nigeria through the lens of the expanded health systems framework
Source: BMJ Glob Health. 2024 Oct 21;9(10):e014610. doi: 10.1136/bmjgh-2023-014610 (PMC11499808; doi:10.1136/bmjgh-2023-014610)
Supplement: online supplemental file 1 [file bmjgh-9-10-s001.pdf]

Glossary of terms

Terms	Abbreviation	Meaning
Community health system	CHS	A community health system (CHS) encompasses local actors, relationships, and processes that promote health in communities and households, distinct from formal health systems.
Formal health provider	FHP	Health professionals operating within officially recognized healthcare institutions or systems.
Informal health provider	IHP	Healthcare providers who operate outside of formal healthcare institutions, often in community-based or non-traditional settings.
Informal health actors	IHA	We used informal health actors as an umbrella term to refer to individuals or groups who provide or advocate for healthcare services outside of formal healthcare systems or structures, often in community settings and without formal training or regulation.
Formal health actors	FHA	Individuals or organizations recognized and sanctioned within the formal healthcare system.
Ward development committees	WDC	WDCs are local community members tasked with organizing and overseeing health and development initiatives at the ward level.
Local Health Representatives	LHR	See village health committees
patent and proprietary medicine vendors	PPMV	They are people authorized to sell over-the-counter medicines and certain non-prescription drugs in Nigeria.
Community Health Influencers and Promoter	CHIP	They work at households to provide counselling and

		create demand for the utilization of PHCs.
Community Health Officers	CHO	A senior community health practitioner in Nigeria.
Officer-in-Charge	OIC	An appointed health worker who serves as the administrative head of a primary health facility.
Community Health Extension Workers	CHEW	They are trained by the college of health technology to provide preventive, curative and rehabilitative health services to community.
Faith-Based Organisations	FBOs	They are groups or institutions affiliated with a particular religious faith or denomination, which undertake various social activities like humanitarian aid.
Voluntary Community Mobilisers	VCM	They are individuals who actively engage and mobilize community members for various health initiatives, with little or no compensation.
Community Based Organisations	CBO	They are grassroots groups formed by community members to address local needs and concerns.
Village health Committees	VHC	They are part of the local governance structure that is meant to maintain accountability for community health.
