# Supplementary material for: Examining the roles and relationships of actors in community health systems in Nigeria through the lens of the expanded health systems framework
Source: BMJ Glob Health. 2024 Oct 21;9(10):e014610. doi: 10.1136/bmjgh-2023-014610 (PMC11499808; doi:10.1136/bmjgh-2023-014610)
Supplement: online supplemental file 2 [file bmjgh-9-10-s002.pdf]

The roles of actors in the community health system

	Actor category	Key actors	Expanded Health System Building Blocks								
**s/ n**			Service delivery	Household production of health & Social determina nts of health	Health workforce	Communit y organisatio ns	Societal partnershi p	Medical products, vaccines and technology	Financing	Leadership and governance	Information , learning and accountabil ity
1	Household-level caregivers	Household Heads and members		1. Use local herbs to treat members. 2. Provide medical advice to neighbour s 3. Organise sanitation 4. Learn local medicine from household heads.		1. Receive health informatio n and act on it.					1. Make household data available
2	Providers	Formal Providers – *Medical Doctors, Midwives, Nurses, CHWs, JCHWs, Health Technicians, Laboratory Scientists;*	1. Provide health services to househol ds. 2. Refer patients to other providers, including IHP.	1. Give health advise to household s.	1. Formal providers are engaged in health facilities.						1. Collect MNCH data from the community

		Environmental officers	3. Go for outreach								
		Informal Providers – *PMVs, TBAs, and Traditional healers (i.e., bonesetters, herbalists, healers)*	1. Provide health services to households.<br>2. Refer patients to Formal providers and IHPs<br>3. Work closely with Formal providers.								
		Volunteers - *Co-Group, and VCM*				1. Move from house to house to mobilise health seekers.					1. Collect MNCH data from the community
3	Organizational intermediaries	NGOs/Agencies - *WHO; Unicef; Red Cross, United Nations.*			1. Organize training of IHP and FP.<br>2. Recruit IHP as volunteers.	1. Contact community leaders to pioneer a project.	1. Initiates partnerships with IHPs, FPs and community leaders.	1. Provide medical products	1. Assist in financing health		
		Community based groups and associations: Youth associations;			1. Recommend volunteers to HODs.	1. Identify health challenges and draw the attention	1. Partner to provide medical tools	1. Assist in the distribution of drugs.	1. Community financing of health	1. WDCs introduce programme officers to village heads.	1. Disseminate information to the public.

		Women Associations; Religious Groups; Transport Unions; Farmers Associations; Town Unions; village/district Heads, PG, Traditional Leaders; WDCs; town criers				of government.<br>2. FBOs, and religious leaders announce health programmes in religious grounds.<br>3. Community groups fund health programmes.			through levies.	2. Community leaders find halls for the programmes .3. Town announcers create awareness. Community advocacy to leaders.	
		Local security networks: Hisbah; Security outfits									
4	Government sectors	Health sectors – LGHA, HODs of health,			1. Approve selected health providers<br>2. Screen and accept IHP and volunteer workers.			1. Provide medical products.	1. Assist in health financing.		1. Health spread health information .
		Non-health sectors - *Education; Agriculture; Social*									1. Help spread health information .

		Welfare; Social Development Officers									
5	Local health representatives and political structures	Village Health Committees				1. Monitor providers<br>2. Work towards the success of health programmes.<br>3. Work closely with other community leaders.		1. Monitor medical products.			1. Health in spreading health information.
6	Private* individuals	Philanthropists						Personally fund health programmes.			

*The actor categories developed by Schneider & Lehmann (2016) did not have private individuals. The study included this group because they featured prominently and did not fit into any other group.
